# Supplementary material for: Microbiome Integrity Protects Against Glial-Mediated Tau and Amyloid Pathology Through Circadian and Autophagy Homeostasis
Source: bioRxiv. 2026 May 22:2026.05.20.726549. Preprint. [Version 1] doi: 10.64898/2026.05.20.726549 (PMC13228631; doi:10.64898/2026.05.20.726549)

bioRxiv preprint doi: https://doi.org/10.64898/2026.05.20.726549; this version posted May 22, 2026. The copyright holder for this preprint (which was not certified by peer review) is the author/funder, who has granted bioRxiv a license to display the preprint in perpetuity. It is made available under a CC-BY 4.0 International license.

File type: Supplementary information:

Manuscript Title:

Gut Microbiome Protects Against Tau and Amyloid Pathology by Preserving Autophagy, Lipid, and Circadian Homeostasis in humanized transgenic *Drosophila* AD model

Kishore Madamanchi[1*], Srinath Gurrala[1], John Watson[1], Girish Melkani[1*]

[1]University of Alabama at Birmingham.

[2]UAB Nathan Shock Center, Birmingham, AL 35294

Email: girishmelkani@uabmc.edu

bioRxiv preprint doi: https://doi.org/10.64898/2026.05.20.726549; this version posted May 22, 2026. The copyright holder for this preprint (which was not certified by peer review) is the author/funder, who has granted bioRxiv a license to display the preprint in perpetuity. It is made available under a CC-BY 4.0 International license.

Figure 1_SI

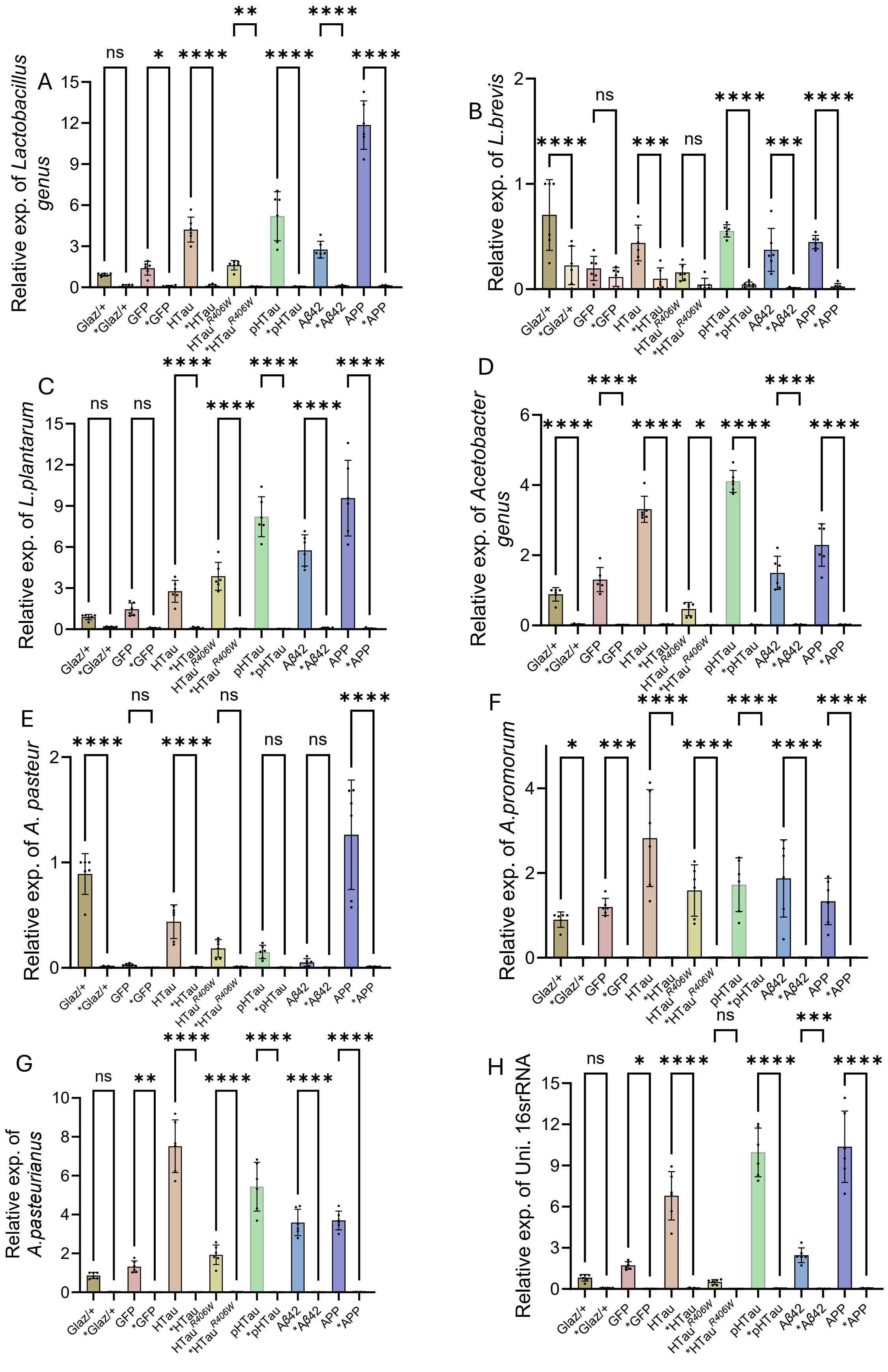

bioRxiv preprint doi: https://doi.org/10.64898/2026.05.20.726549; this version posted May 22, 2026. The copyright holder for this preprint (which was not certified by peer review) is the author/funder, who has granted bioRxiv a license to display the preprint in perpetuity. It is made available under a CC-BY 4.0 International license.

SI. Figure 1. Represents the comparison of most abundant microbial population in fly gut between conventional control (CC) and axenic antibiotic (AA) flies carrying AD transgenes expressed under *Glaz-Gal4* glial cell specific driver at 3 weeks age. (A). *Lactobacillus* genus AA flies showed a significant reduction in HTau, pTau, aβ42, APP (p<0.0001) and GFP, HTau^{R406W} (p<0.01) compared to CC flies. (B). *L. brevis* showed significant decrease in AA flies (p<0.0001) compared to CC flies in *Glaz*/+, HTau, pTau, aβ42, and APP flies. (C). *L. plantarum* Significant reduction in GFP, HTau (p<0.001), HTau^{R406W}, pTau, aβ42, APP (p<0.0001) in AA flies compared to CC. (D). *Acetobacter* in AA flies showed a significant reduction in *Glaz*/+, GFP, HTau, pTau, aβ42, APP (p<0.0001) and HTau^{R406W} (p<0.01) compared to CC flies. (E). *A. pasteur* in *Glaz*/+, HTau, and APP flies in AA showed significant decrease compared to CC flies. *A. promorum Glaz*/+, GFP (p<0.01) and HTau, HTau^{R406W}, pTau, aβ42, APP (p<0.0001) in AA flies decreased significantly compared to CC flies. (G). *A. pasteurianus* showed significant reduction in GFP (p<0.01), HTau, HTau^{R406W}, pTau, aβ42, APP (p<0.0001) in AA flies compared to CC flies. (H). 16 rRNA quantification showed significant reduction in GFP (p<0.01), HTau, pTau, aβ42, APP (p<0.0001) in AA flies compared to CC flies. n=3, p-values * <0.05, ** <0.01, *** < 0.001, **** <0.0001.

bioRxiv preprint doi: https://doi.org/10.64898/2026.05.20.726549; this version posted May 22, 2026. The copyright holder for this preprint (which was not certified by peer review) is the author/funder, who has granted bioRxiv a license to display the preprint in perpetuity. It is made available under a CC-BY 4.0 International license.

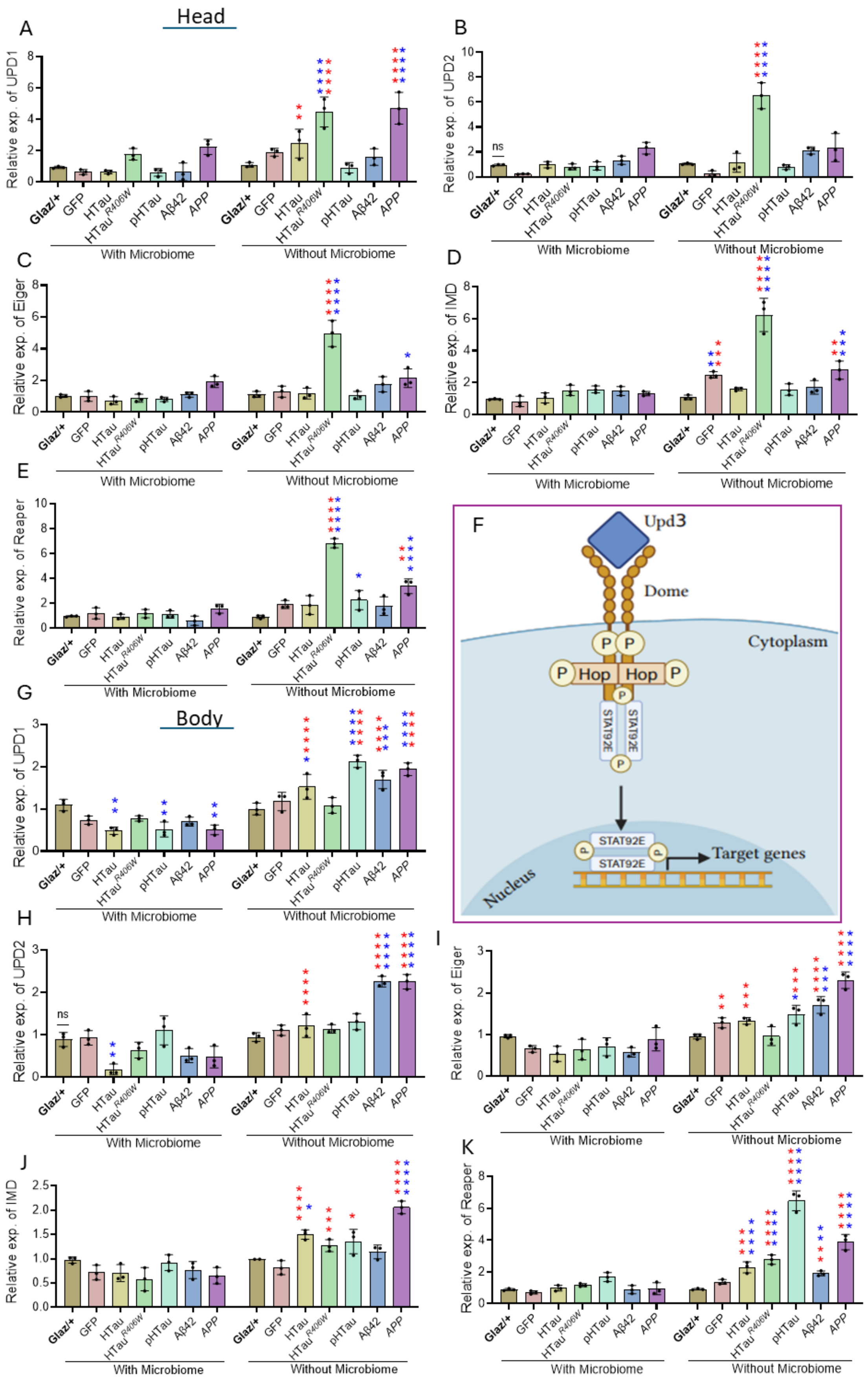

bioRxiv preprint doi: https://doi.org/10.64898/2026.05.20.726549; this version posted May 22, 2026. The copyright holder for this preprint (which was not certified by peer review) is the author/funder, who has granted bioRxiv a license to display the preprint in perpetuity. It is made available under a CC-BY 4.0 International license.

SI. Figure 2. Represents cytokine (*Upd*1, and 2), *Eiger* (*TNFα* homolog) its downstream target *Imd* and cell death marker *Reaper* expression in head and body regions. (A). *Upd*1 expression have no significant difference in CC flies, in AA HTau^{R406W}, and APP (p<0.0001) showed a significant difference compared to controls. HTau (p<0.01), HTau^{R406W}, and APP (p<0.0001) showed significant increase compared to CC flies. (B). *Upd*2 expression found to be increased only in HTau^{R406W} (p<0.0001) compared to control and CC counter part in AA flies. (C). *Eiger,* showed increased expression in HTau^{R406W} (p<0.0001), and APP (p<0.1) compared to control in AA flies and HTau^{R406W} (p<0.0001) compared to CC flies. (D). *Imd* increase in GFP (p<0.01), HTau^{R406W} (p<0.0001), GFP (p<0.001), HTau^{R406W} (p<0.0001), and APP (p<0.01) compared CC flies. (E). *Reaper* gene expression increased in AA HTau^{R406W}, APP (p<0.0001), and pTau (p<0.05) compared controls and HTau^{R406W} (p<0.0001), APP (p<0.01) compared to CC flies in head region. (F). Schematic representation of inflammatory signaling through *Upd 3-Stat92e* pathway. (G). *Upd* 1 expression decreased in HTau, pTau and APP (p<0.01) in CC flies, and HTau (p<0.05), pTau, APP (p<0.0001) and aβ42 (p<0.001) in AA flies compared to controls. In AA flies HTau, pTau, aβ42 and APP (p<0.0001) showed significant increase compared to CC flies. (H). *Upd 2* showed significant decrease in HTau (p<0.01) in CC flies, aβ42 and APP (p<0.0001) in AA flies compared to controls. In AA flies HTau, aβ42 and APP (p<0.0001) showed a significant increase compared to CC flies.(I). *Eiger* expression increased in pTau (p<0.05), aβ42 (p<0.001) and APP (p<0.0001) compared to control in AA flies, and Compared to CC flies GFP (p<0.01), HTau, pTau (p<0.001), aβ42 and APP (p<0.0001) increased significantly in AA flies. (J). Imd upregulated in AA HTau (p<0.05) and APP (p<0.0001) compared to control, also HTau, APP (p<0.0001), HTau^{R406W} (p<0.001), and pTau (p.0.05) showed significant increase in AA flies compared to CC flies. (K). *Reaper* significantly upregulated in AA, HTau, HTau^{R406W}, pTau, APP (p<0.0001) and aβ42 (p<0.01) compared to controls. Also, compared to CC flies HTau (p<0.001), HTau^{R406W}, pTau, APP (p<0.0001) and aβ42 (p<0.01) increased significantly in AA flies. n=3, p-values * <0.05, ** <0.01, *** < 0.001, **** <0.0001.

bioRxiv preprint doi: https://doi.org/10.64898/2026.05.20.726549; this version posted May 22, 2026. The copyright holder for this preprint (which was not certified by peer review) is the author/funder, who has granted bioRxiv a license to display the preprint in perpetuity. It is made available under a CC-BY 4.0 International license.

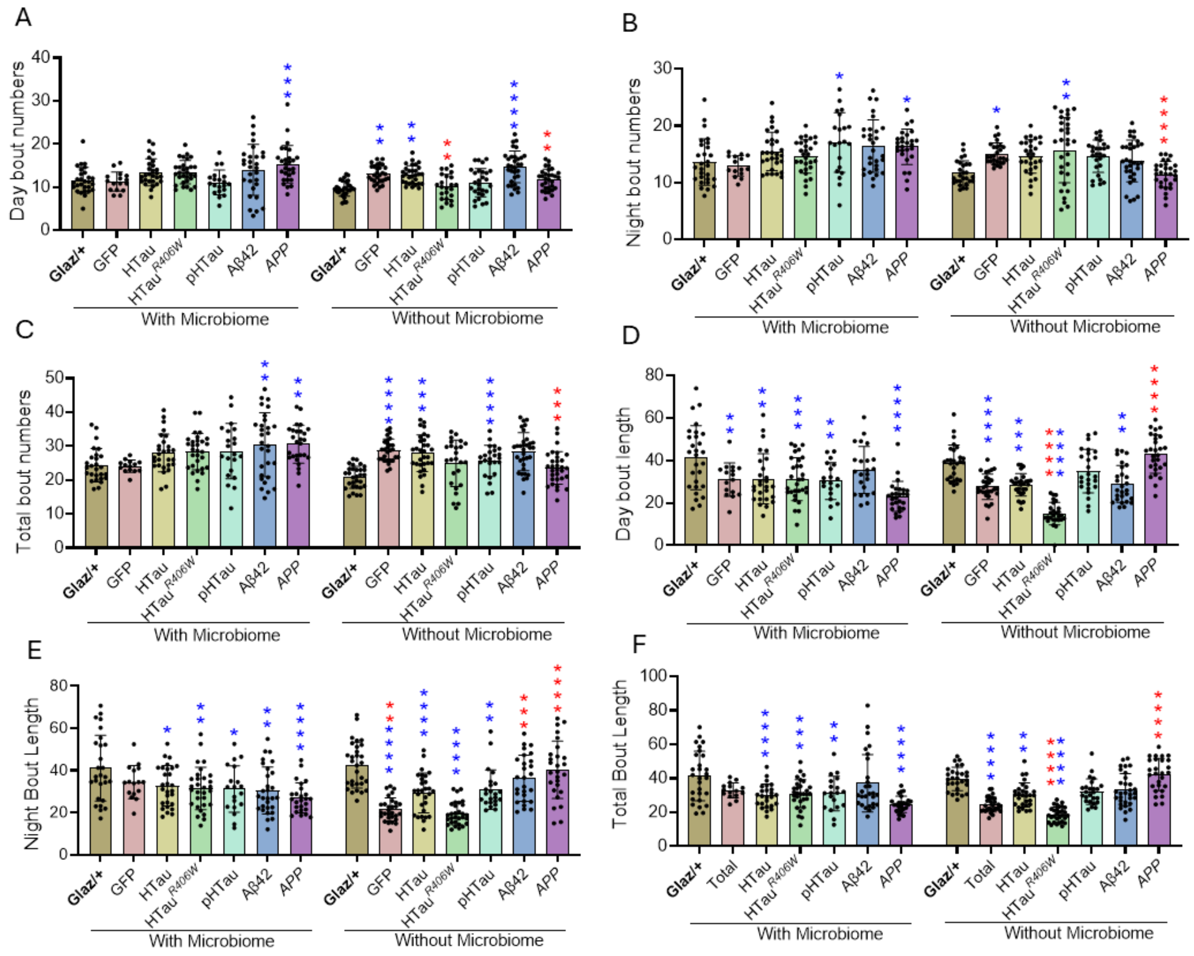

SI. Figure 3. Represents the bout numbers, and bout length. in CC and AA flies at 3 weeks age. (A). Day sleep bout numbers increased in APP flies compared to control in CC, and in AA flies GFP, HTau ($p<0.01$) and aβ42 ($p<0.0001$) compared to controls, (B). Night sleep bout numbers increased in pTau and APP ($p<0.05$) in CC flies, In AA flies GFP ($p<0.05$), HTau^{R406W} ($p<0.01$) compared to controls. In APP of AA flies significant increase observed ($p<0.0001$) compared to CC flies. (C). Total sleep bout numbers increase in aβ42, APP ($p<0.0001$) of CC flies, in AA flies GFP, pTau ($p<0.0001$) and HTau ($p<0.001$) compared to controls. In APP of AA flies significant increase observed ($p<0.001$) compared to CC flies. (D) Day sleep bout length decreased in GFP, HTau, and pTau ($p<0.01$), HTau^{R406W} ($p<0.001$) and APP ($p<0.0001$) compared to controls. In AA flies GFP, HTau^{R406W} ($p<0.0001$), HTau, aβ42 ($p<0.01$) decreased significantly compared to control. In AA flies HTau^{R406W} ($p<0.0001$) significantly decreased, and APP ($p<0.0001$) significantly increased compared to CC flies. (E). Night sleep bout length decreased in HTau, pTau ($p<0.05$), HTau^{R406W} , aβ42 ($p<0.01$) and APP ($p<0.0001$) in CC flies compared to control. In AA flies GFP, HTau, HTau^{R406W} ($p<0.0001$), and pTau ($p<0.01$) decreased compared to controls. In AA GFP ($p<0.01$), decreased and aβ42 ($p<0.001$), APP ($p<0.0001$) significantly increased compared to CC flies. (F). Total sleep bout length Decreased in HTau, APP ($p<0.0001$), HTau^{R406W} ($p<0.001$), and pTau ($p<0.01$) compared to control I n CC flies. In AA flies GFP, HTau^{R406W} ($p<0.0001$), and HTau ($p<0.01$) decreased significantly compared to control in AA flies. In HTau^{R406W} ($p<0.001$) decreased significantly and in APP ($p<0.0001$) increased significantly compared to CC flies. n=3, p-values * <0.05, ** <0.01, *** < 0.001, **** <0.0001.

bioRxiv preprint doi: https://doi.org/10.64898/2026.05.20.726549; this version posted May 22, 2026. The copyright holder for this preprint (which was not certified by peer review) is the author/funder, who has granted bioRxiv a license to display the preprint in perpetuity. It is made available under a CC-BY 4.0 International license.

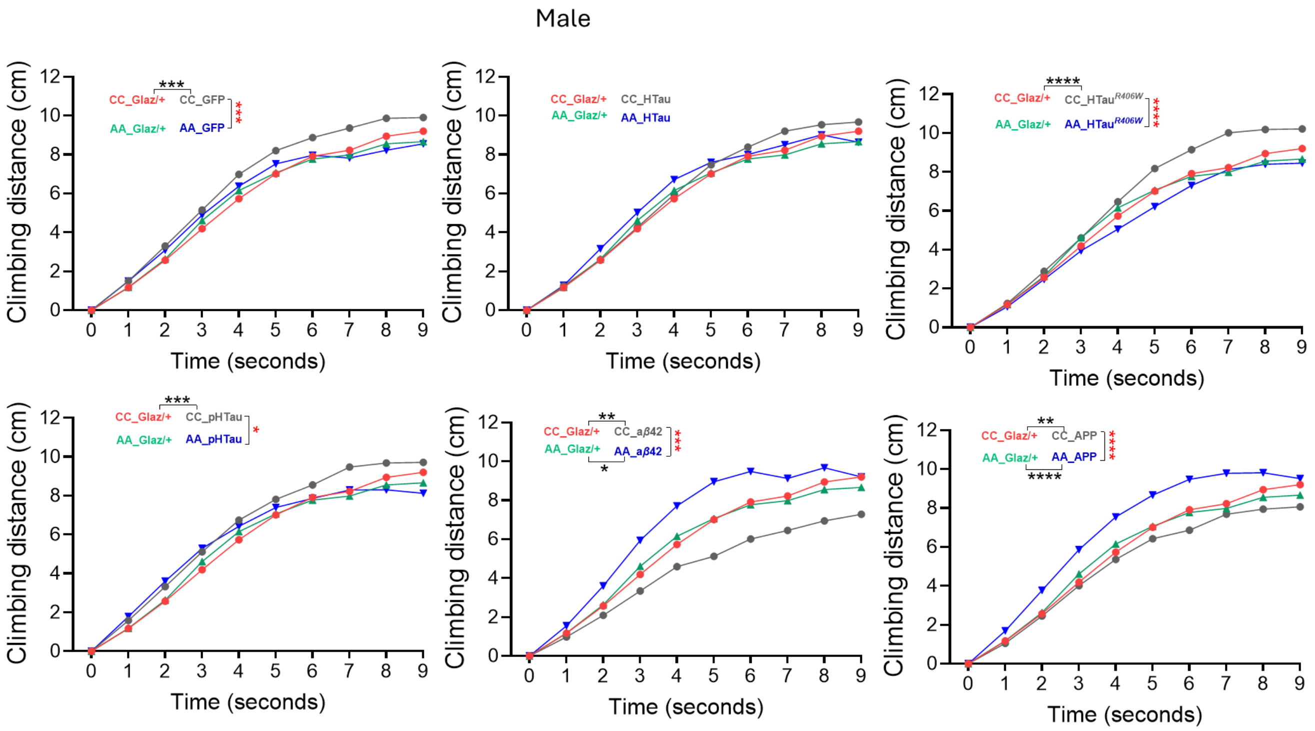

SI. Figure 4. Male fly geotaxis performance during CC and AA condition from 0 to 9 seconds. Climbing distance showed no significant difference between AA and CC control flies. Whereas in AA flies GFP and Glaz/+ showed reduced ($p<0.0001$, $p<0.001$) compared CC GFP. HTau found no significant difference. R406W in CC showed a significant increase ($p<0.0001$, $p<0.0001$) compared to Glaz/+ and AA R406W. pHTau in CC showed increased compared to AA and Glaz/+ ($p<0.03$, $p<0.001$). AB42 in AA showed a significant increase ($p<0.001$, $p<0.008$) compared to Ab42 and Glaz/+ in CC. We also seen significant difference between AA Glaz/+ and AA AB42 ($p<0.04$). In APP, AA flies showed a significant increase ($p<0.0001$, $p<0.0001$) compared to AA, Glaz/+ and CC APP, in addition significant difference observed between CC Glaz/+ and CC APP ($p<0.007$). Two-way ANOVA followed by Sidak's multiple comparisons test used for statistical analysis. n=3, p-values * <0.05, ** <0.01, *** < 0.001, **** <0.0001.

bioRxiv preprint doi: https://doi.org/10.64898/2026.05.20.726549; this version posted May 22, 2026. The copyright holder for this preprint (which was not certified by peer review) is the author/funder, who has granted bioRxiv a license to display the preprint in perpetuity. It is made available under a CC-BY 4.0 International license.

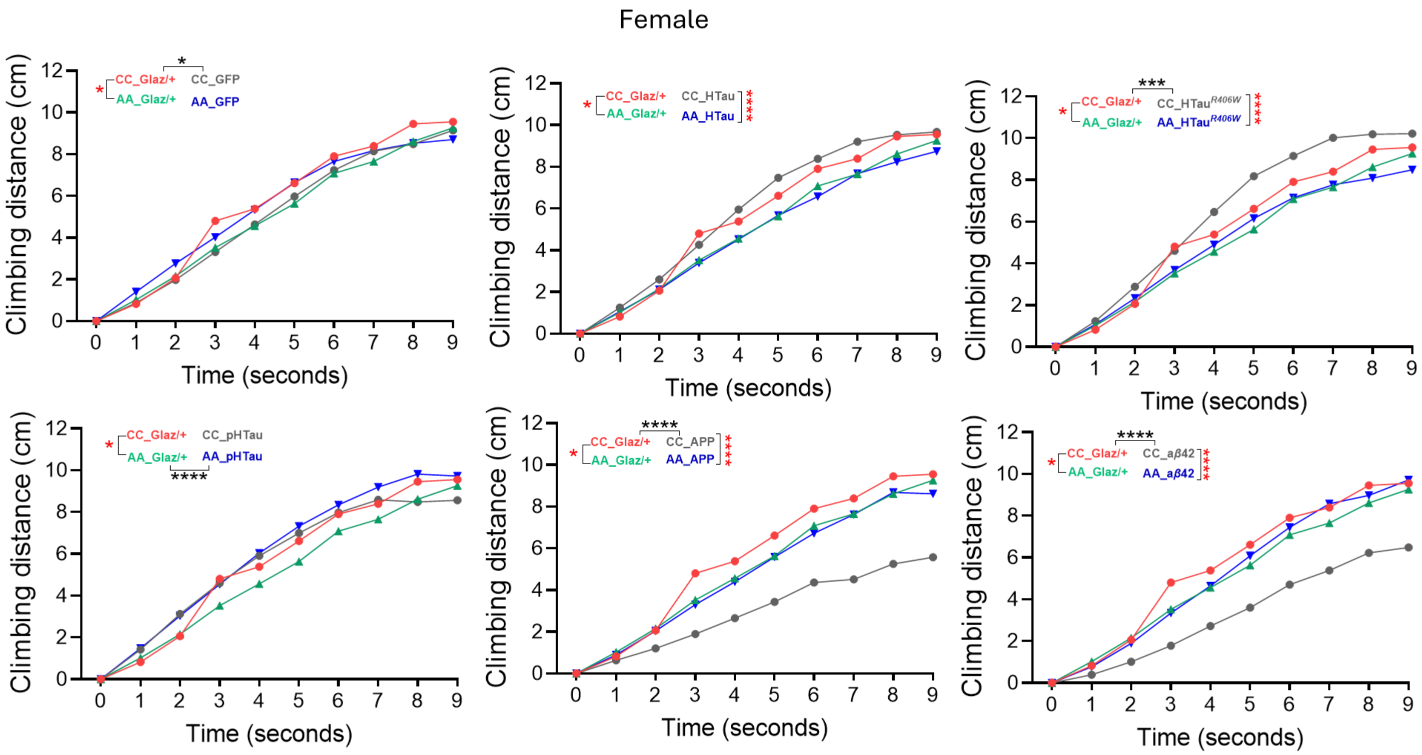

SI. Figure 5. Female fly geotaxis performance during CC and AA condition from 0 to 9 seconds. Climbing distance showed significant difference ($p<0.02$) between AA and CC control (Glaz/+) flies. In CC condition Glaz/+ showed significant increase ($p<0.04$) compared to GFP. In HTau CC showed significant increase compared to AA ($p<0.0001$). R406W in CC showed a significant increase ($p<0.001$, $p<0.0001$) compared to Glaz/+ and AA R406W. pHTau in AA showed significant increase compared to AA Glaz/+ ($p<0.0001$). AB42 in CC showed a significant reduction ($p<0.0001$, $p<0.0001$) compared to Glaz/+ and AA AB42. APP in CC showed a significant reduction ($p<0.0001$, $p<0.0001$) compared to Glaz/+ and AA APP flies. Two-way ANOVA followed by Sidak's multiple comparisons test used for statistical analysis. n=3, p-values * <0.05, ** <0.01, *** < 0.001, **** <0.0001.

bioRxiv preprint doi: https://doi.org/10.64898/2026.05.20.726549; this version posted May 22, 2026. The copyright holder for this preprint (which was not certified by peer review) is the author/funder, who has granted bioRxiv a license to display the preprint in perpetuity. It is made available under a CC-BY 4.0 International license.

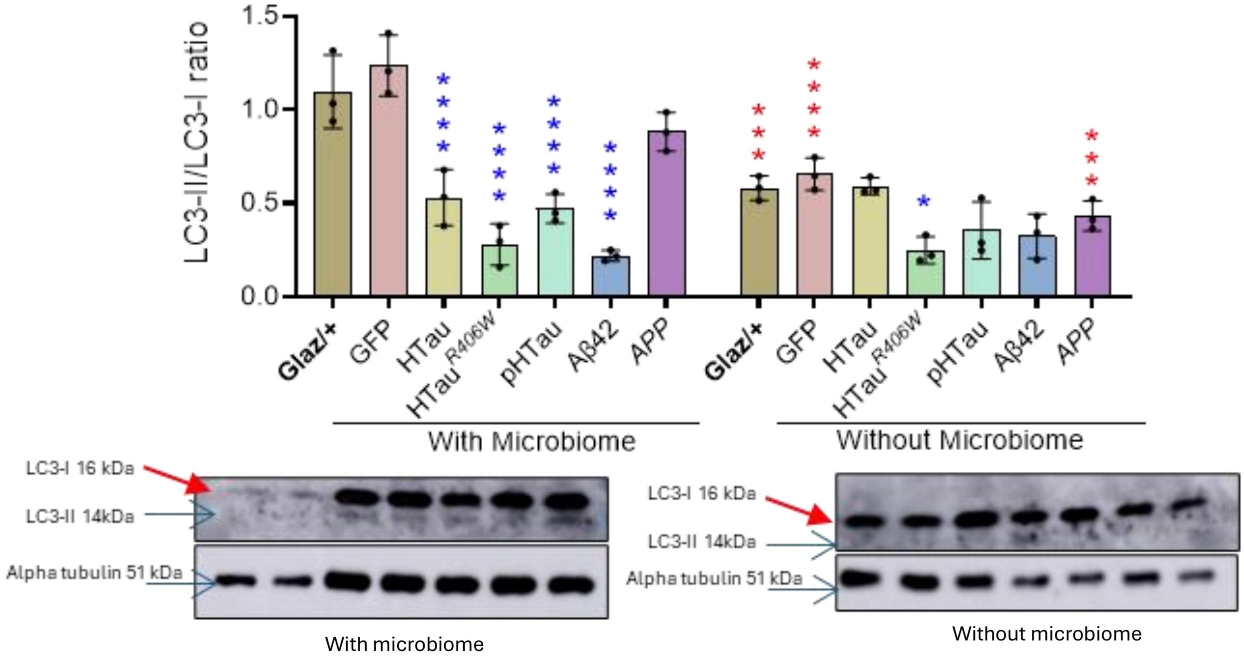

SI. Figure 6. Representing the quantification data of LC3-II vs LC3-I ratio in transgenic flies. We compared the ration between two groups (with and without microbiome), also with in the groups we compared control vs transgenes. Our study showed a significant decrease in LC3-II expression in AD transgenes (with microbiome) compared to control and found a drastic reduction in axenic (without microbiome) condition. LC3 band intensity was normalized with alpha tubulin to understand the relative abundance of protein loading into the gels. Then we calculated the LC3-II/LC3-I ratio between genotypes and groups. Compared to Glaz/+, HTau, R406W, pHTau and AB42 showed a significant decrease ($p<0.0001$) in CC and R406W in AA ($p<0.03$) in each group. In AA flies Glaz/+ ($p<0.001$), GFP ($p<0.0001$), APP ($p<0.001$) showed a significant decrease compared to CC condition. n=3, p-values * <0.05, ** <0.01, *** < 0.001, **** <0.0001.

bioRxiv preprint doi: https://doi.org/10.64898/2026.05.20.726549; this version posted May 22, 2026. The copyright holder for this preprint (which was not certified by peer review) is the author/funder, who has granted bioRxiv a license to display the preprint in perpetuity. It is made available under a CC-BY 4.0 International license.

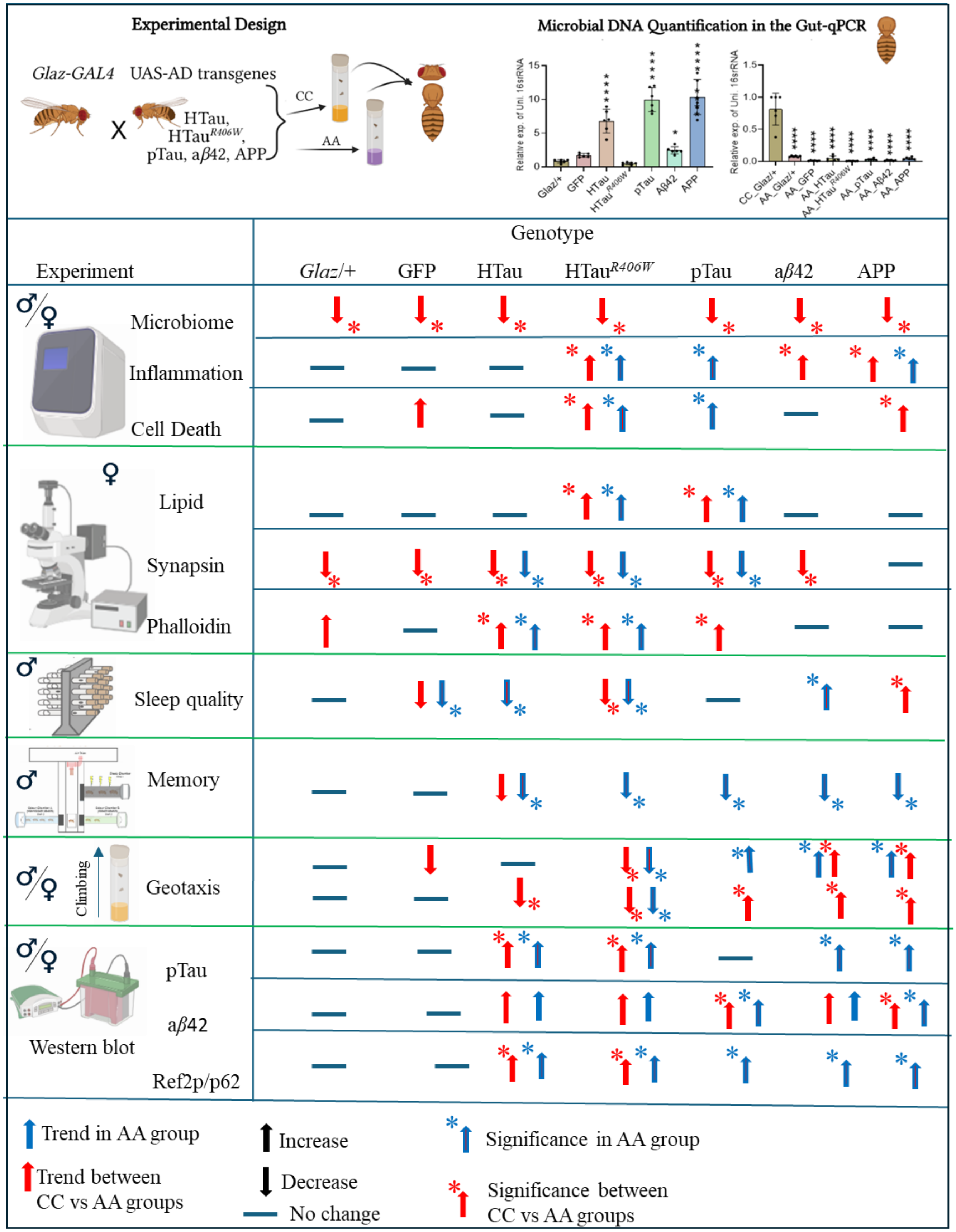

bioRxiv preprint doi: https://doi.org/10.64898/2026.05.20.726549; this version posted May 22, 2026. The copyright holder for this preprint (which was not certified by peer review) is the author/funder, who has granted bioRxiv a license to display the preprint in perpetuity. It is made available under a CC-BY 4.0 International license.

Supplementary figure 7: Summary Figure: Schematic representation of Work plan, and gut microbiome quantification using CC, AA flies. Experimental procedures including real time PCR for inflammatory, cell death markers and microbiome quantification. immunofluorescence analysis for lipid, synapsin and phalloidin staining. Sleep activity analysis with *Drosophila* Activity monitoring System (DAMS). Memory/Olfaction assay with T-maze. Geotaxis analysis and western blot analysis. Figure represents axenic fly data, compared with control (blue arrow) and between groups (CC vs AA, red arrow). Star represents significance. Up-arrow (Significant increase) Down-arrow (Significant decrease).

bioRxiv preprint doi: https://doi.org/10.64898/2026.05.20.726549; this version posted May 22, 2026. The copyright holder for this preprint (which was not certified by peer review) is the author/funder, who has granted bioRxiv a license to display the preprint in perpetuity. It is made available under a CC-BY 4.0 International license.

Raw Data for Western blots

AB42_WB_for Glaz_AD_CC_AA _Head

Glaz _AD_**CC**_ Head

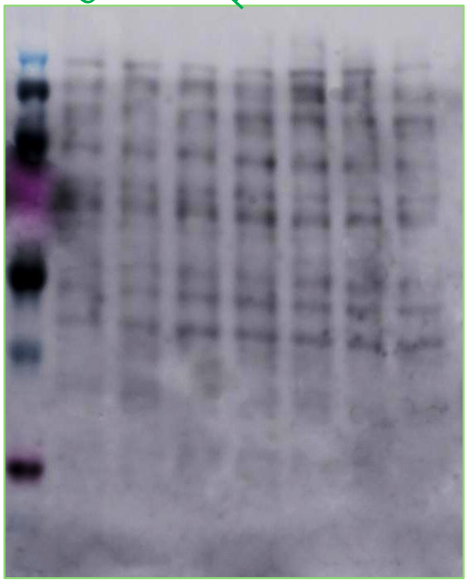

Glaz _AD_**AA**_ Head

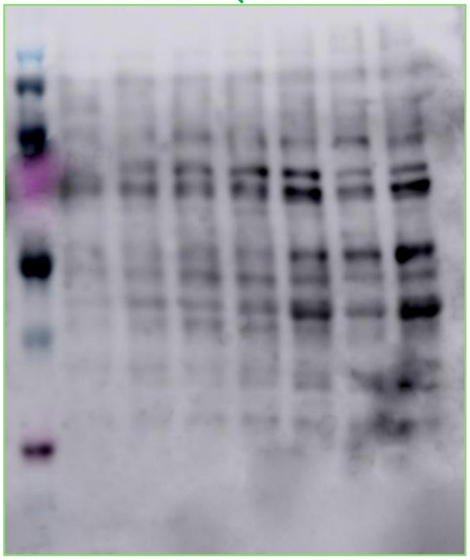

Tau _WB for Glaz_AD_AA_CC_Head

Glaz _AD_**CC**_ Head

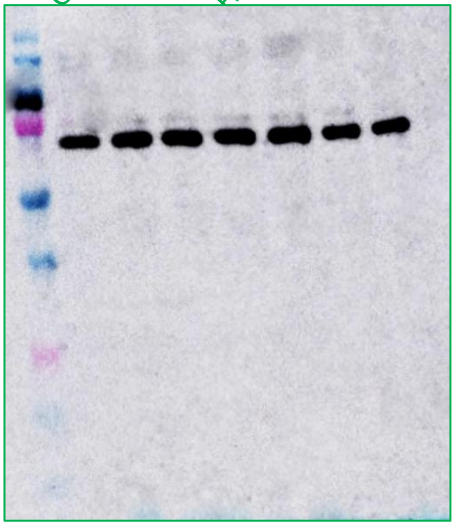

Glaz _AD_**AA**_ Head

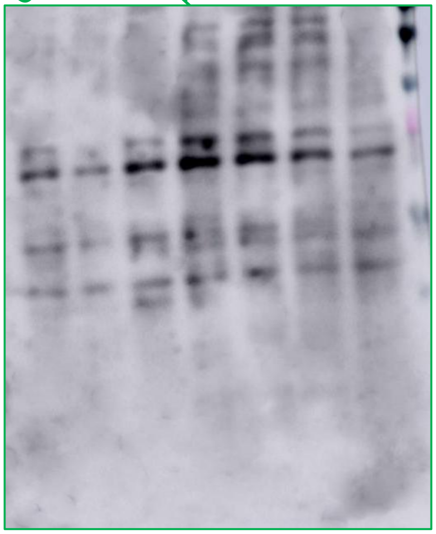

bioRxiv preprint doi: https://doi.org/10.64898/2026.05.20.726549; this version posted May 22, 2026. The copyright holder for this preprint (which was not certified by peer review) is the author/funder, who has granted bioRxiv a license to display the preprint in perpetuity. It is made available under a CC-BY 4.0 International license.

pTau _WB for Glaz_AD_CC_AA_Head

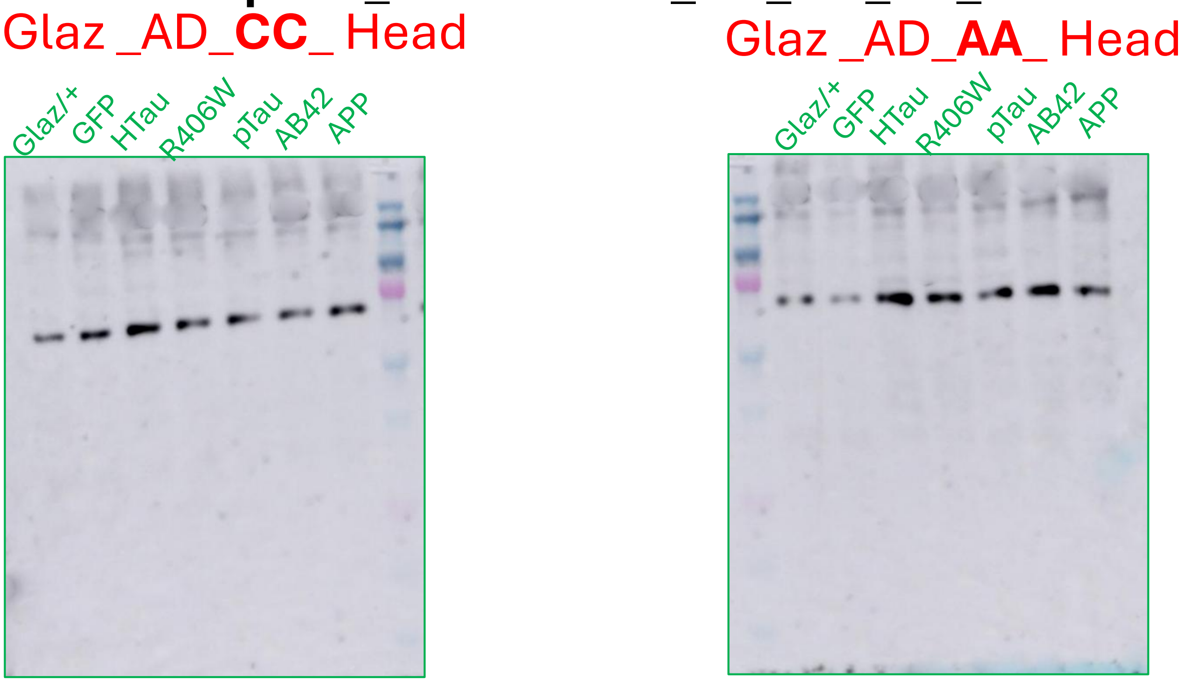

Synaptotagmin 1 _WB for Glaz_AD_AA_CC_Head

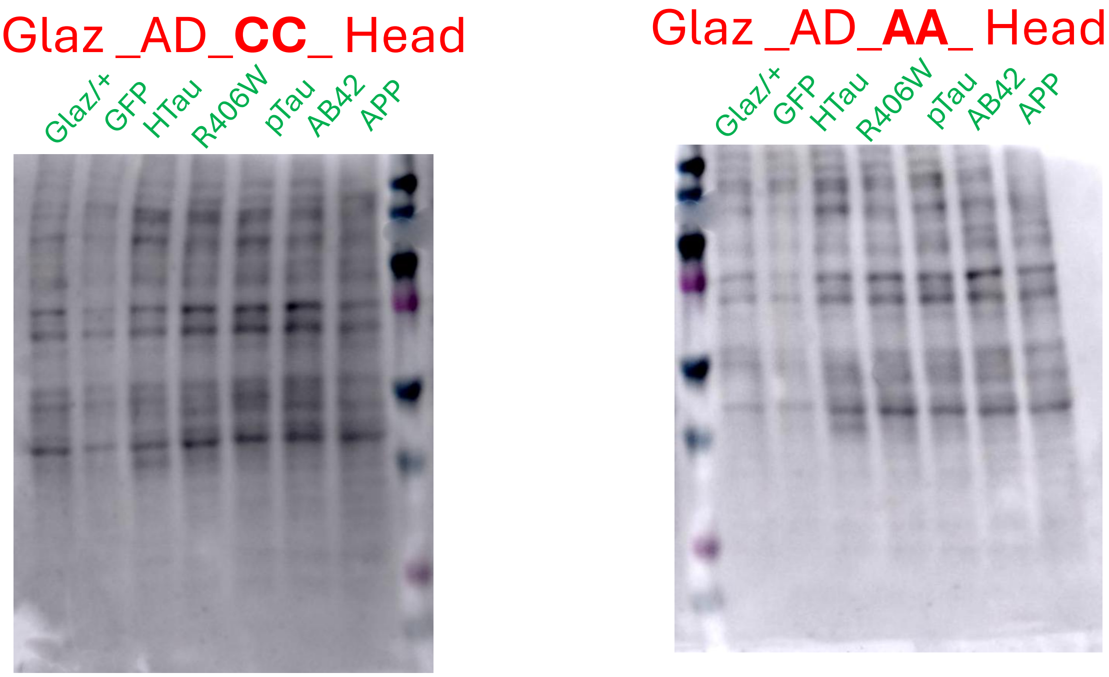

bioRxiv preprint doi: https://doi.org/10.64898/2026.05.20.726549; this version posted May 22, 2026. The copyright holder for this preprint (which was not certified by peer review) is the author/funder, who has granted bioRxiv a license to display the preprint in perpetuity. It is made available under a CC-BY 4.0 International license.

AMPK_WB for Glaz_AD_AA_CC_Head

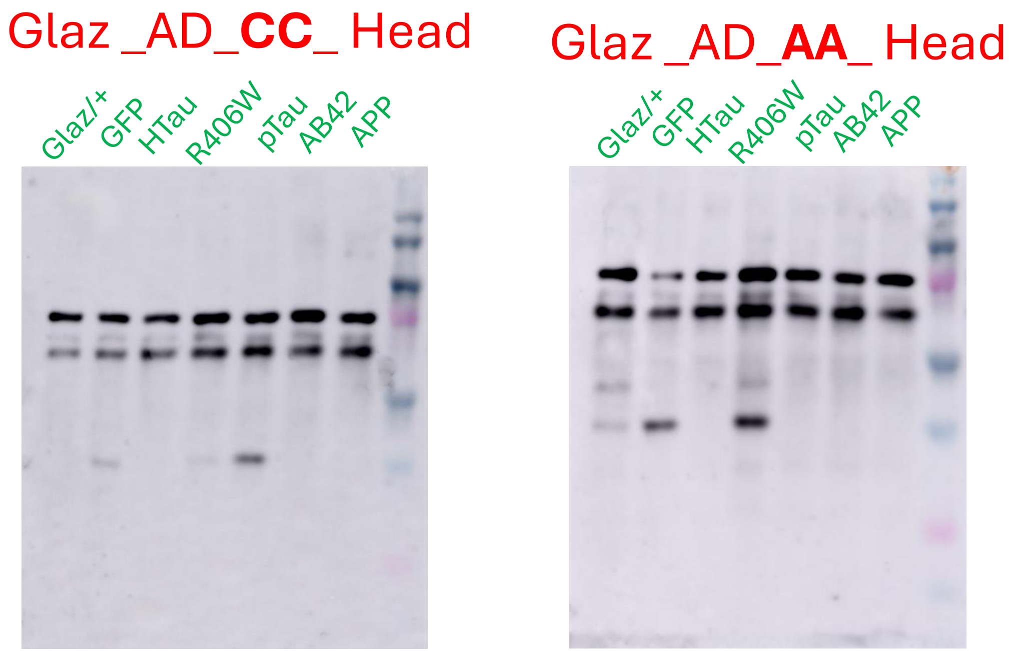

pAMPK_WB for Glaz_AD_AA_CC_Head

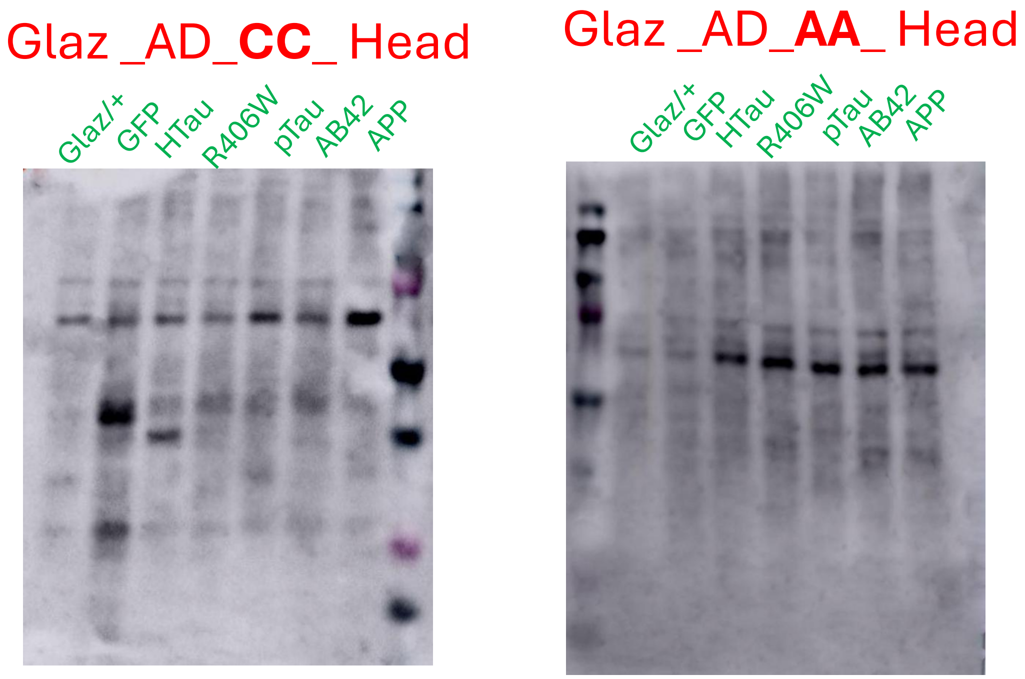

bioRxiv preprint doi: https://doi.org/10.64898/2026.05.20.726549; this version posted May 22, 2026. The copyright holder for this preprint (which was not certified by peer review) is the author/funder, who has granted bioRxiv a license to display the preprint in perpetuity. It is made available under a CC-BY 4.0 International license.

Ubiquitin_WB for Glaz_AD_AA_CC_Head

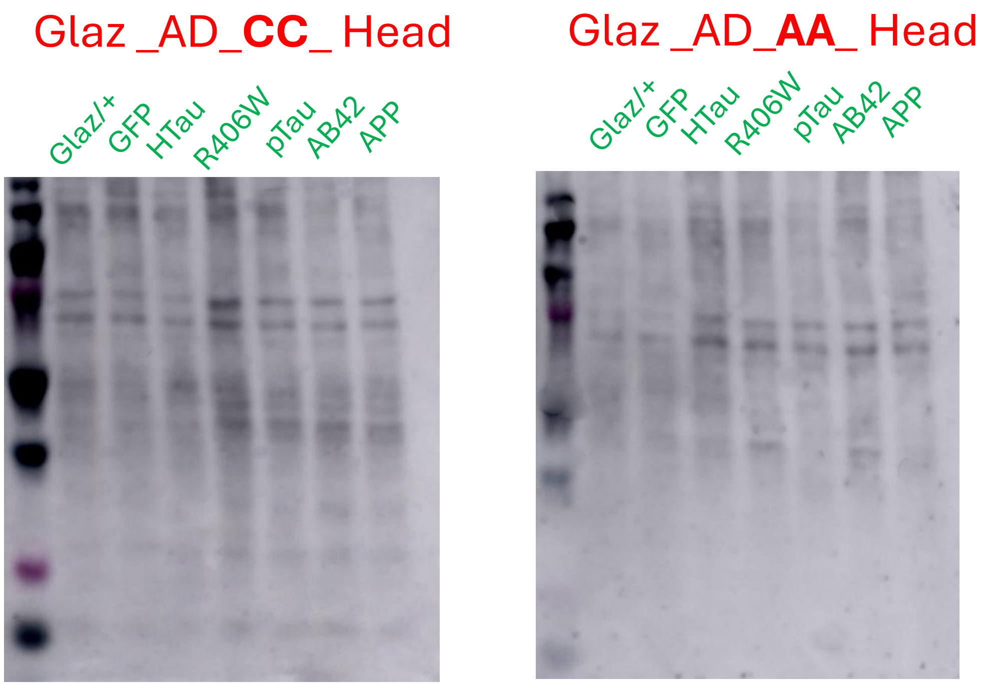

Ref2p (p62) WB for Glaz_AD_AA_CC_Head

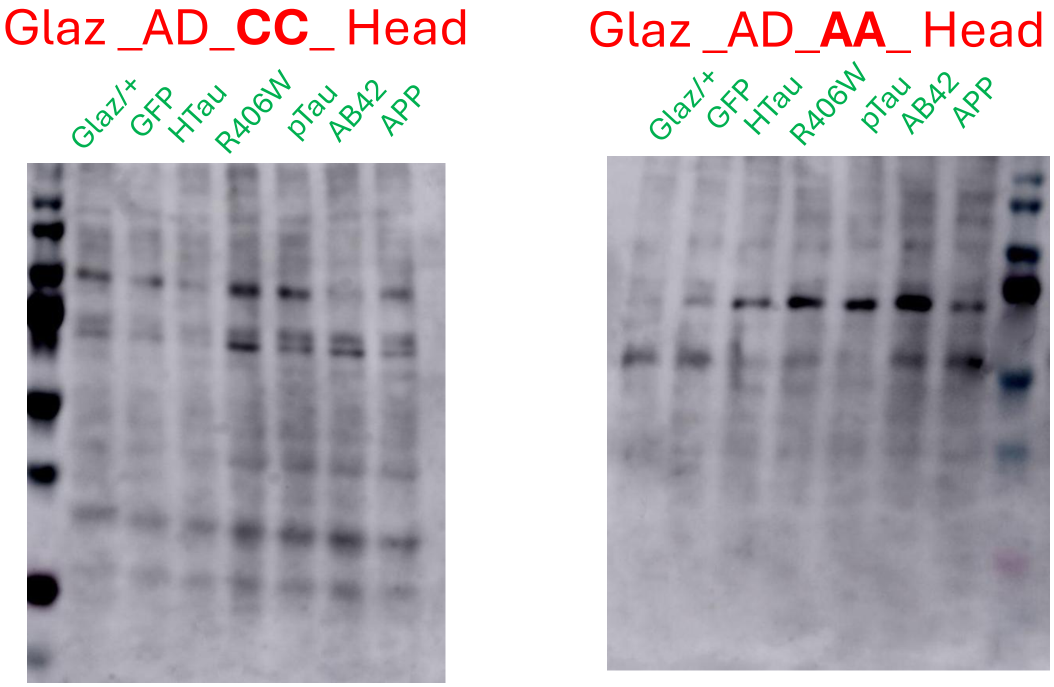

bioRxiv preprint doi: https://doi.org/10.64898/2026.05.20.726549; this version posted May 22, 2026. The copyright holder for this preprint (which was not certified by peer review) is the author/funder, who has granted bioRxiv a license to display the preprint in perpetuity. It is made available under a CC-BY 4.0 International license.

LC3-I/II_WB for Glaz_AD_AA_CC_Head

Glaz _AD_**CC**_ Head

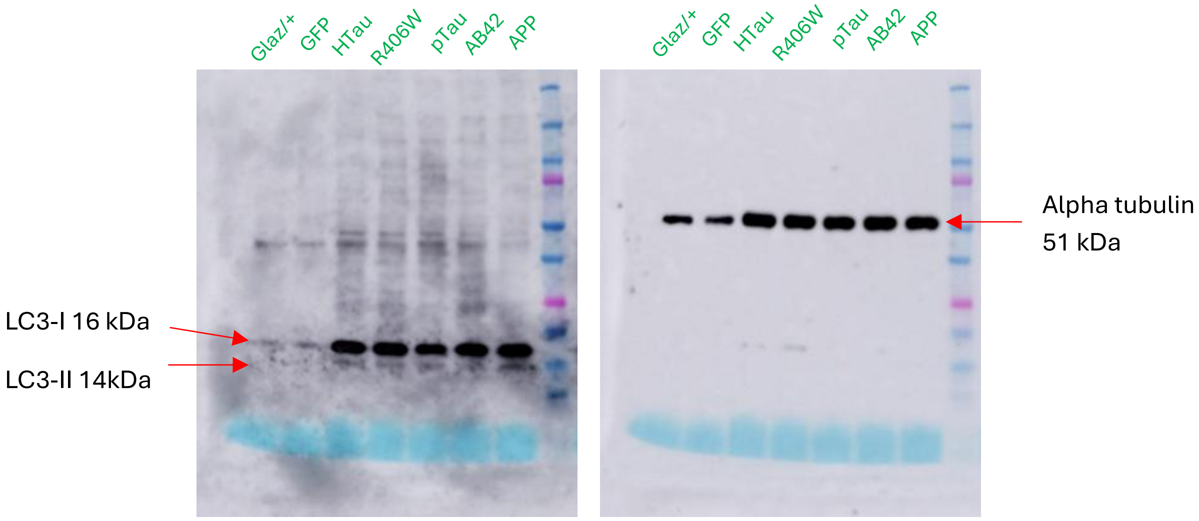

Glaz _AD_**AA**_ Head

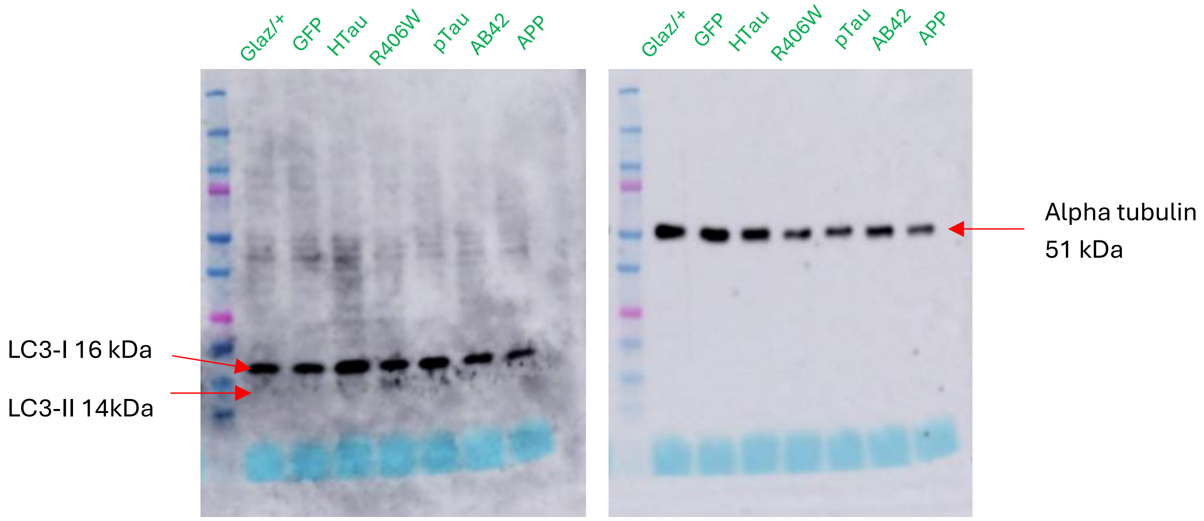

Supplement: Supplement 1 [file NIHPP2026.05.20.726549v1-supplement-1.pdf]
